# Trends in Pneumococcal and Bacterial Meningitis in Brazil from 2007 to 2019

**DOI:** 10.3390/vaccines11081279

**Published:** 2023-07-25

**Authors:** Cintia Irene Parellada, Ariane de Jesus Lopes de Abreu, Marina G. Birck, Carolina Zampirolli Dias, Thais das Neves Fraga Moreira, Guilherme Silva Julian, Paula de Mendonça Batista, Juan Carlos Orengo, Ana Luiza Bierrenbach

**Affiliations:** 1 MSD Brazil, São Paulo 04583-110, Brazil; thais.moreira@merck.com (T.d.N.F.M.); paula.de.mendonca.batista@merck.com (P.d.M.B.); 2 IQVIA Brazil, São Paulo 04719-002, Brazil; arianeabreu.rj@gmail.com (A.d.J.L.d.A.); marinagabriela.birck@iqvia.com (M.G.B.); carolina.zampirolli@iqvia.com (C.Z.D.);; 3 MSD (IA) LLC, Guaynabo 00966, Puerto Rico; juan_orengo@merck.com; 4 Instituto de Ensino e Pesquisa, Hospital Sírio-Libanês, São Paulo 01308-050, Brazil; albierrenbach@yahoo.com.br

**Keywords:** meningitis, invasive pneumococcal disease, vaccination, Brazil, epidemiology

## Abstract

The pneumococcal conjugate vaccination (PCV) was introduced into the Brazilian Childhood National Immunization Program in 2010; however, universal pneumococcal vaccination for older adults has not been implemented yet. Our aim is to evaluate the trends in pneumococcal meningitis incidence and case fatality rate (CFR) across all age groups from 2007 to 2019 using data from the National Surveillance System. The pre-PCV (2007–2009) and post-PCV (2011–2019) periods were compared; changes in incidence and CFR were assessed by joinpoint regression. Additional analyses of bacterial meningitis were performed to compare the patterns and trends. Over the 13-year period, 81,203 and 13,837 cases were classified as bacterial and pneumococcal meningitis, respectively. *S. pneumoniae* was the main etiological agent of bacterial meningitis in adults aged ≥50 years and the most lethal in all age groups. In the post-PCV period, a 56.5% reduction in the average incidence was seen in pneumococcal meningitis in the pediatric population. In contrast, there was an increasing trend among adults. The CFR for pneumococcal and bacterial meningitis remained stable in most age groups during the study period. These findings highlight the value of expanding pneumococcal vaccination policies, including vaccines that provide better indirect protection from children to adults and broadening vaccination to older adults.

## 1. Introduction

Bacterial meningitis represents a public health challenge with a high case fatality rate (CFR) and complications that may include permanent sequelae [1,2]. Although it can be caused by several types of bacteria, *Streptococcus pneumoniae*, *Haemophilus influenzae*, and *Neisseria meningitidis* are the predominant pathogens [3]. The incidence and mortality rates for bacterial meningitis vary by region and age group [4]. Worldwide, pneumococcal meningitis is the leading type of bacterial meningitis, associated with more than 44,500 deaths and 2,720,000 years of life lost in 2019 [5].

In Brazil, meningitis is a compulsory notification disease and all suspected cases should be reported to the National Information System for Notifiable Diseases (*Sistema de Informação de Agravos de Notificação*, SINAN), regardless of etiology [6,7]. Moreover, there is a passive laboratory-based surveillance system for invasive pneumococcal disease, comprising a network of state reference public health laboratories and private hospitals that refer isolates to the national reference laboratory, Adolfo Lutz Institute, for serotyping, antimicrobial susceptibility testing, and molecular typing [8]. The SINAN and Adolfo Lutz Institute databases are independent and do not have any identifier code that links their data.

The childhood immunization schedule of the Brazilian National Immunization Program contains vaccines targeting the main pathogens responsible for bacterial meningitis. In 1999, the *Haemophilus influenzae* type b vaccine was introduced into the National Immunization Program, followed by both the 10-valent pneumococcal vaccine (PCV10) and the meningococcal C conjugate vaccine in 2010 [9]. High sustained vaccination coverage rates for these vaccines (≥80%) were achieved between 2011 and 2019 [10]. Since 1999, pneumococcal vaccines have been offered to institutionalized older adults as part of public national campaigns or for patients at high risk of pneumococcal disease; however, the coverage rate in this setting is estimated to be <15% [11,12,13,14].

After childhood PCV10 introduction, changes in pneumococcal disease epidemiology have been reported across all age groups worldwide due to *S. pneumoniae* serotype replacement, the emergence of new non-vaccine serotypes, and changing antimicrobial susceptibility [15,16,17,18]. Surveillance systems play a crucial role in evaluating the impact of these vaccination programs and monitoring local epidemiological trends [19]. Therefore, this study aimed to evaluate trends in the incidence and CFR of pneumococcal meningitis across all age groups in Brazil by using national disease surveillance data over a 13-year period. Moreover, further analysis of bacterial meningitis was performed to compare patterns and trends in the same period.

## 2. Materials and Methods

### 2.1. Study Design and Data Sources

This was a nationwide population-based descriptive study with a time-series component using secondary data on meningitis surveillance in Brazil between 2007 and 2019. To assess the number of cases and deaths, data on meningitis cases were extracted from the SINAN de-identified database in July 2021 [20]. Projected population estimates from the 2010 national census were obtained from the Brazilian Institute of Geography and Statistics (*Instituto Brasileiro de Geografia e Estatística*—IBGE) and used to calculate pneumococcal and bacterial meningitis incidence rates [21]. National pediatric vaccination coverage rates of PCV10 were extracted from the National Immunization Program Information System (SI-PNI), available through the online database of the Brazilian Health Ministry (DATASUS) [10].

### 2.2. Study Population and Case Definitions

All suspected and confirmed cases of meningitis have to be reported to the local health authority by any professionals working in the area of healthcare services and surveillance, including public and private diagnostic laboratories [6,7]. A suspected case was defined as any individual of any age with fever, headache, vomiting, neck stiffness, other meningeal sign, seizures, petechial rash, and/or altered mental state [7]. For infants under 9 months old, irritability (persistent crying) and bulging fontanelles were considered. A confirmed case of meningitis was defined as a suspected case with the presence of at least one of the following criteria: laboratory confirmation by culture, polymerase chain reaction (PCR), or latex agglutination test; or bacterioscopy of cerebrospinal fluid (CSF) with the presence of the pathogen; or CSF chemical or cytology findings suggestive of bacterial or viral meningitis; or fulfillment of specific clinical/epidemiologic criteria [7].

All confirmed cases of meningitis were classified in SINAN into the following ten etiological categories: (1) meningococcemia, (2) meningococcal meningitis, (3) meningococcal meningitis with meningococcemia, (4) tuberculous meningitis, (5) meningitis by other bacteria, (6) unspecified meningitis, (7) aseptic meningitis, (8) meningitis due to another etiology, (9) *Haemophilus* meningitis, and (10) pneumococcal meningitis [7]. All confirmed cases of the categories meningococcemia, meningococcal meningitis, meningococcal meningitis with meningococcemia, *Haemophilus* meningitis, pneumococcal meningitis, and meningitis by other bacteria were included in this study. Other meningitis categories were excluded, including tuberculous meningitis, due to its subacute course and specific epidemiology. Meningococcemia, meningococcal meningitis, and meningococcal meningitis categories were grouped as meningococcal disease. For this study, bacterial meningitis was defined as all cases of meningococcal disease, *Haemophilus* meningitis, pneumococcal meningitis, and meningitis by other bacteria excluding tuberculous meningitis. The overall study population included all age groups stratified into the following groups: <1 year, 1–4 years, 5–17 years, 18–49 years, 50–59 years, and ≥60 years. Under Brazilian law, adults were defined as persons aged 18 years or older and older adults as persons aged 60 years or older [22].

### 2.3. Data Analysis

Vaccination coverage was based on the number of doses administered to people in a specified target population divided by the respective total estimated target population [10]. The absolute number of confirmed meningitis cases and deaths was described by type of bacterial meningitis and age groups for the entire period. CFR was calculated by dividing the deaths due to meningitis by the number of confirmed cases of meningitis in the period, expressed as percentage.

Annual incidence rates were determined separately for all bacterial meningitis and for pneumococcal meningitis, using cases as numerators and the annual IBGE population as denominators [21]. Incidence rates were calculated per 100,000 inhabitants per year and by age group. Ninety-five percent confidence intervals (95% CIs) were estimated for the rates, assuming a Poisson distribution.

The time trend analyses focus on population-level effects of pediatric pneumococcal vaccination based on average exposure in the pre- and post-vaccination periods and disease outcomes (pneumococcal and bacterial meningitis incidence and CFR). To compare pre-PCV10 (2007–2009) and post-PCV10 (2011–2019) vaccination periods, the percentage of change and 95% CI (Poisson distribution) between the average incidence rate and CRF of pneumococcal and bacterial meningitis for each period were calculated. We considered 2010 a transition year and excluded it from this analysis because high vaccination uptake started to be achieved in 2011. Changes were considered statistically significant if the 95% CI did not cross zero.

To identify significant trend changes in the incidence rate and CFR of pneumococcal and bacterial meningitis, joinpoint regression analysis was used. The algorithm applied in this analysis tests whether a multi-segmented line is a significantly better fit than a straight or less-segmented line. Line segments are joined at points called joinpoints [23]. It was used the grid search method, in which the ideal number of joinpoints was selected using a Monte Carlo permutation test (4499 permutations) with a minimum of zero joinpoints (one line segment) and a maximum number of two joinpoints (three line segments) for the entire study period (2007–2019). In addition to best-fit line segments, expressed as annual percentage change (APC), the average APC (AAPC) was determined for the following three prespecified fixed intervals: (a) the entire study period (2007–2019), (b) the entire post-PCV10 period (2011–2019), and (c) the late post-PCV10 period (2016–2019). AAPC is a summary measure of the trend over these pre-specified fixed intervals, if the AAPC segment lies entirely within the line or joint segment line, it will be equal to the APC for that segment. The late post-PCV10 period was based on establishment of a mature PCV program, where disease and colonization by vaccine serotypes contained in the PCV are largely controlled, usually after PCV use for at least 5 years with uptake >70% [24]. Time trends were considered statistically significant when APC or AAPC had a *p*-value < 0.05.

Analyses were performed using R version 4.1.0 (R Foundation for Statistical Computing, Vienna, Austria). The joinpoint regression analysis was performed using Join Point version 4.9.0.0 (Statistical Research and Applications Branch, National Cancer Institute, Rockville, MD, USA) [25].

## 3. Results

### 3.1. Vaccination Coverage

The national vaccination coverage for the three doses of PCV10 and meningococcal C vaccine rose from around 25% in 2010 to an average of 91.4% throughout 2011–2019. The *Haemophilus influenzae* vaccine had an average vaccination coverage of 80.7% throughout the study period (Table S1).

### 3.2. Descriptive Statistics for Bacterial Meningitis Cases and Deaths

From 2007 to 2019, 259,065 confirmed cases of meningitis were reported to SINAN, and 99.1% (256,799) had a defined etiology (Table S2). Of those with defined etiology, 81,203 (31.6%) cases were classified as bacterial meningitis. The bacterial meningitis with the greatest number of cases and deaths was meningitis by other bacteria, followed by meningococcal disease, pneumococcal meningitis, and *Haemophilus* meningitis (Table 1). During the study period, *N. meningitidis* was the most common bacterial etiology in age groups under 50 years, followed by *S. pneumoniae.* In the age groups ≥50 years, *S. pneumoniae* was the main etiology. Pneumococcal meningitis had the highest CFR among all bacterial meningitis categories and across all age groups (Table 1).

**Figure 1:**
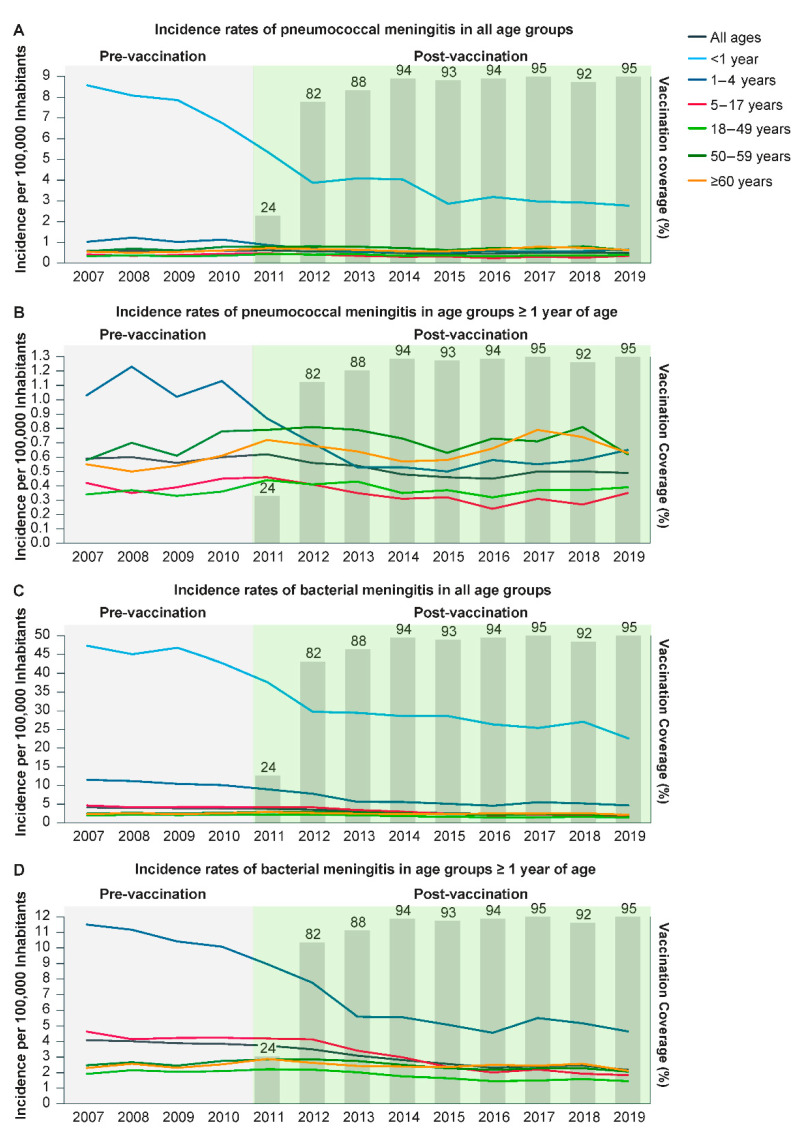
Annual incidence rates of pneumococcal and bacterial meningitis per 100,000 individuals by year and age group, Brazil, 2007–2019. (**A**) Pneumococcal meningitis incidence rate by age group. (**B**) Pneumococcal meningitis incidence rate in age groups ≥ 1 year of age. (**C**) Bacterial meningitis incidence rate by age group. (**D**) Bacterial meningitis incidence rate in age groups ≥1 year of age. Infant pneumococcal vaccination coverage (%) is displayed in gray bars starting at the introduction of this vaccine in the Brazilian National Immunization Program in 2010.

The annual incidence rate of pneumococcal and bacterial meningitis decreased over the study period, mainly in the age groups under 5 years old (Figure 1 and Table S3).

### 3.3. Time-Series Analysis

**Figure 2:**
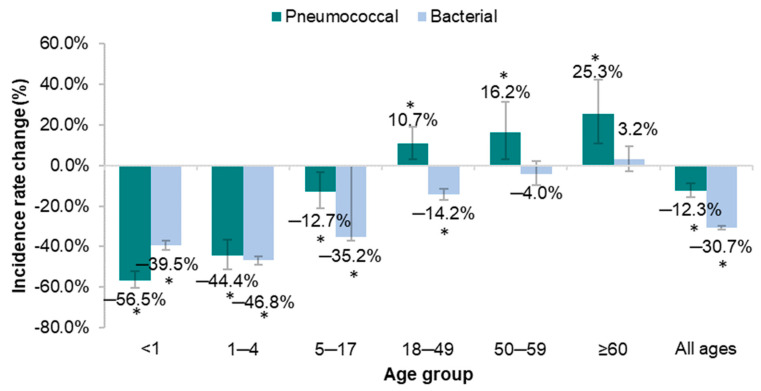
Incidence rate change in pneumococcal and bacterial meningitis in the before-after analysis (comparison between pre-PCV10 (2007–2009) and post-PCV10 (2011–2019) periods). The change is given as a percentage. The error bars represent the 95% CI. *p* < 0.05 marked with an asterisk. Changes were considered statistically significant if the 95% CI did not cross zero. See also Table S4.

In the before-after analysis, there was a 12.3% reduction of pneumococcal meningitis in the overall population (Figure 2). The greatest reductions in the pneumococcal meningitis incidence rates were reported among infants aged <1 year (from 8.2 to 3.6 cases/100,000), followed by children aged 1–4 years (from 1.1 to 0.6 cases/100,000), and those aged 5–17 years (from 0.4 to 0.3 cases/100,000; Table S4). Among adults, there was a significant increase in pneumococcal meningitis incidence rates in the post-PCV10 period compared to the pre-PVC10 period, mainly in the age groups 50–59 years (from 0.6 to 0.7 per 100,000) and ≥60 years old (from 0.5 to 0.7 per 100,000; Figure 2 and Table S4).

For bacterial meningitis, there was a decrease in the incidence rate in the post-PCV10 period for all age groups except those ≥60 years old (Figure 2). However, it was not statistically significant (95% CI −2.7; 9.6; Table S4). The greatest reductions were reported for infants <1 year of age (from 46.4 to 28.0 cases/100,000), children aged 1–4 years (from 11.0 to 5.9 cases/100,000), and children and adolescents aged 5–17 years (from 4.3 to 2.8 cases/100,000) (Figure 2 and Table S4).

**Figure 3:**
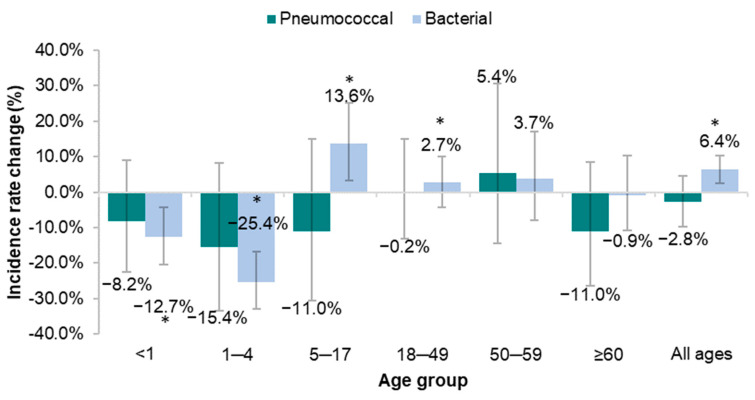
Case fatality rate change in pneumococcal and bacterial meningitis in the before-after analysis (comparison between pre-PCV10 (2007–2009) and post-PCV10 (2011–2019) periods). The change is given as a percentage. The error bars represent the 95% CI. *p* < 0.05 is marked with an asterisk. Changes were considered statistically significant if the 95% CI did not cross zero. See also Table S4.

The CFR of pneumococcal meningitis showed a decrease in most age groups (except for ages 50–59) in the post-PCV10 period compared to the pre-PCV10 period (Figure 3 and Table S5), but none of the results were statistically significant (all 95% CIs crossed zero). For bacterial meningitis, the CFR decreased significantly in infants <1 year of age (−12.7%; 95% CI −20.4, −4.1) and children 1–4 years old (−25.4%; 95% CI −32.9, −16.9) in the post-PCV period. However, a statistically significant increase was seen in those aged 5–17 years old (13.6%; 95% CI 3.0, 25.1; Figure 3 and Table S5).

**Figure 4:**
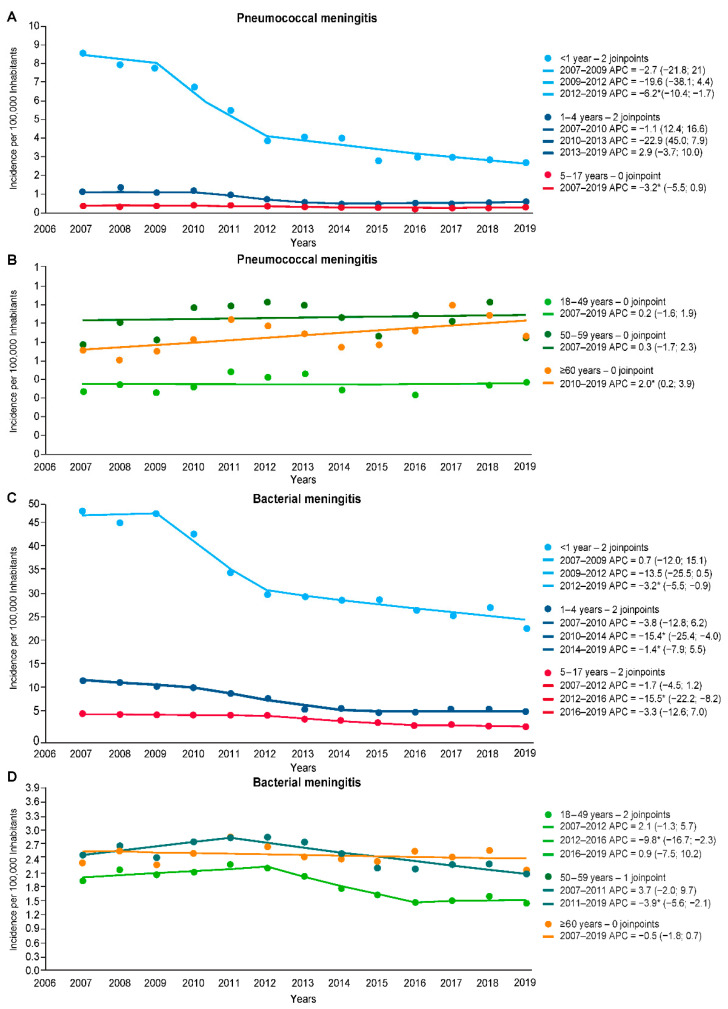
Joinpoint regression analysis of incidence rates of pneumococcal and bacterial meningitis by age group, 2007–2019, Brazil. (**A**) Pneumococcal meningitis rates in the pediatric population. (**B**) Pneumococcal meningitis rates in adults. (**C**) Bacterial meningitis rates in the pediatric population. (**D**) Bacterial meningitis rates in adults. * *p*-value < 0.05.

In the time trend analysis using joinpoint regression, the pneumococcal meningitis incidence in the overall population was relatively stable from 2007 to 2011, then reduced by 6.8% per year until 2015 and increased by 2.4% from 2015 to 2019 (Table 2). However, none of the three segments analyzed by time trend inflection was statistically significant. Among infants aged <1 year, there was a slight decline in the magnitude of change in the late PCV10 period (AAPC −6.2; 95% CI −10.4, −1.7) compared to the entire post-PCV10 period (AAPC −8.0; 95 CI 11.5, −4.3). In children aged 1–4 years, there was a statistically non-significant increasing trend in the pneumococcal meningitis incidence in the late PCV10 period (AAPC 2.9; 95% CI −3.7, 10). Among children and adolescents aged 5–17 years, pneumococcal meningitis incidence reduced by 3.2% by year throughout the entire study. Among adults, the pneumococcal meningitis incidence rates were relatively stable over the study period, except for older adults, who showed a 2.0% increase per year during the same period (Table 2). Figure 4A,B illustrates the different segments of the joinpoint analyses of pneumococcal meningitis incidence.

For bacterial meningitis, the incidence in the overall population was significantly reduced by 8.9% per year from 2011 to 2016, followed by a non-significant annual reduction of 1.0% from 2016 to 2019 (Table 2). Most age groups showed a significant decreasing trend in bacterial meningitis incidence starting in 2010–2012. During 2016–2019, a stationary trend was observed for most age groups, except for children aged <1 year (AAPC −3.2; 95% CI −5.5, −0.9) and adults aged 50–59 years (AAPC −3.9; 95 CI −5.6, −2.1; Table 2). Figure 4C,D illustrates the different segments of the joinpoint analyses of bacterial meningitis incidence.

**Figure 5:**
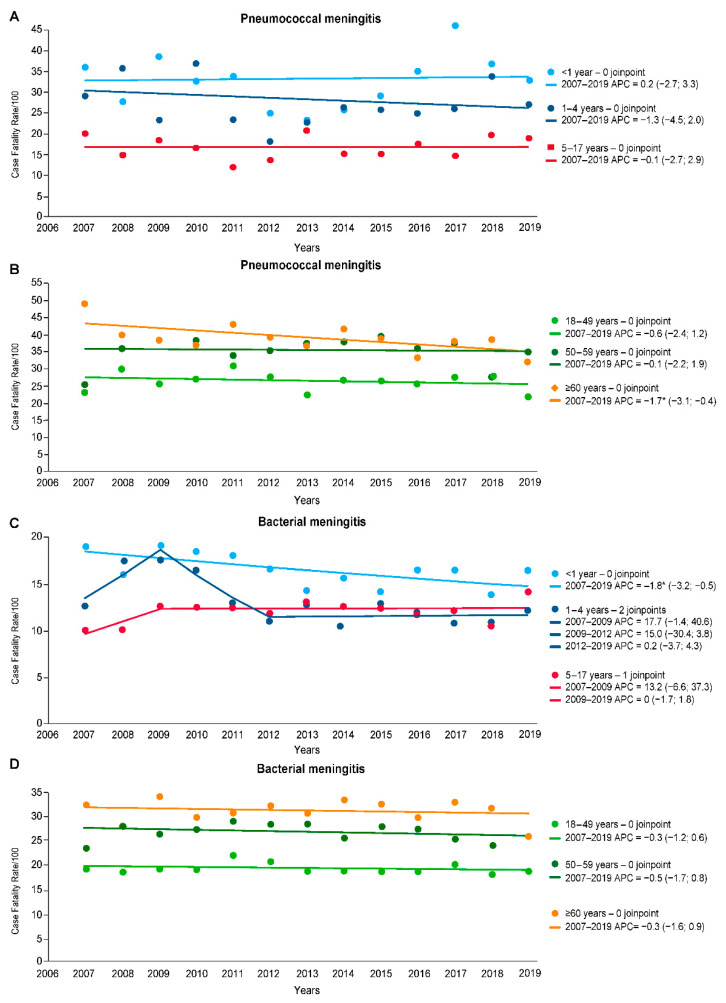
Joinpoint regression analysis of case fatality rate of pneumococcal and bacterial meningitis by age group, 2007–2019, in Brazil. (**A**) Pneumococcal meningitis rates in the pediatric population. (**B**) Pneumococcal meningitis rates in adults. (**C**) Bacterial meningitis rates in the pediatric population. (**D**) Bacterial meningitis rates in adults. * *p*-value <0.05.

The CFR of pneumococcal and bacterial meningitis remained stable for most age groups during the study period (Table 3). However, for older adults, significant reductions were seen in pneumococcal meningitis, with an average reduction of 1.7% per year (95% CI −3.1, −0.4). For bacterial meningitis, there was a significant CFR reduction of 1.8% per year (95% CI −3.2, −0.5) among infants aged <1 year from 2007 to 2019. Figure 5 illustrates the different segments of the joinpoint analyses of CFR for pneumococcal and bacterial meningitis.

## 4. Discussion

This study analyzed a 13-year period from a nationwide meningitis surveillance database and evaluated the impact of PCV10 on the pneumococcal meningitis epidemiology across all age groups in Brazil. The beneficial effect of PCV10 in reducing pneumococcal meningitis incidence has been shown in the target population eligible for vaccination in the National Immunization Program. However, no indirect effect was seen in adults. The additional analysis of bacterial meningitis allowed us to better characterize pneumococcal meningitis in terms of lethality and changing epidemiology across different age groups.

In our study, while *N. meningitidis* was the most common causative agent of bacterial meningitis overall, *S. pneumoniae* was predominated in the age groups ≥50 years. These findings contrast with data from the United States and other countries in Latin America that reports *S. pneumoniae as* the most common etiology of bacterial meningitis in all age groups after the introduction of routine infant immunization for *H. influenza, N. meningitidis*, and *S. pneumoniae* [26,27].

Like other studies [8,28,29,30,31], we found a substantial reduction in pneumococcal disease in the pediatric population after childhood PCV introduction. In contrast, we observed a significant increase in the mean incidence rate of pneumococcal meningitis in the post-PCV10 introduction period in adults, with an increasing APC of +2.0% in older adults between 2007 and 2019. Even after taking into account a potential lag period by assessing the late post-vaccination time period, the incidence trend continued to increase. This high increasing frequency of pneumococcal meningitis in adults may reflect a sum number of factors, including high rates of pneumococcal infection in the community, lack of herd protection in adults, and no public routine pneumococcal immunization program targeting older adults. Our findings are in line with previous studies in Brazil evaluating pneumococcal disease impact post-PCV introduction that did not support PCV10 indirect protection in older adults [8,32].

Unfortunately, the serotypes involved in meningitis cases are unavailable in the SINAN database. However, it can be inferred that serotype distributions are the same reported by the invasive pneumococcal disease surveillance in Brazil. The 2022 report showed a predominance of serotypes not contained in PCV10 causing meningitis, mainly types 19A (26.2%), 6A/C (13.4%), and 3 (9.1%) [33]. PCV10-type totaled a tiny proportion of pneumococcal meningitis cases, 5.4% for children <5 years and 4.6% for those ≥5 years. In the latter age group, the specific-serotype coverage provided by the 23-valent pneumococcal polysaccharide vaccine was 55.4%, indicating that a substantial burden might be avoided with the introduction of routine pneumococcal vaccination for older adults [33].

Data from the PSERENADE project, including 32 countries using PCV13 and eight countries using PCV10 with well-established routine infant immunization programs and high uptake, found that the prevalence of vaccine-in-use serotypes was lower (≤26% across all ages) than in the pre-PCV era (≥70% in children) [24]. Interestingly, while the percentage of PCV10-type was similar between Brazil and other countries in children <5 years (~5%), the coverage for older children and adults was only 7.6%, compared to 14.9% in other countries [24]. This finding, summed with the increasing incidence trend of pneumococcal meningitis observed in our study, highlights the importance of considering disease incidence and serotype distribution to inform national pneumococcal vaccination policies [24,33].

The optimal vaccination strategy for the protection of the elderly population should consider direct protection based on the burden of the disease, not only in pneumococcal meningitis but also in other clinical presentations. Brazil is an aging country with increasing morbidity and mortality rates per lower respiratory infections, among them, pneumococcal pneumonia [34,35]. Therefore, a combination of direct protection for older adults and herd immunity from infants to adults should be considered. Newer vaccines with broader coverage are expected in the upcoming years; however, this promise should not delay the implementation of public interventions with currently approved vaccines in Brazil [36]. Some options may be PPSV-23 alone or a sequential regimen with PCV13/PCV15. Moreover, a broader spectrum of PCVs containing the most frequent serotypes causing pneumococcal disease in infants has the potential to increase the coverage against these serotypes for this population and provide additional protection through herd immunity for older ages [37].

In our study, the main difference seen between pneumococcal and all bacterial meningitis trends was the increasing incidence trend for pneumococcal meningitis among adults over the study period compared to a downward trend in bacterial meningitis. Although pneumococcal meningitis had the highest CFR among all bacterial meningitis, a reduction of 1.7% each year was seen in older adults over the study period. This decrease may be related to better management of the cases, improved diagnostic and laboratory practices, or even earlier diagnoses with better prognoses [38]. Otherwise, an increase was observed among children and adolescents aged 5–17 years for bacterial meningitis during 2012–2019. This increase seems to be related to the increase in the number of cases of meningococcal disease with serogroup C, mainly in the adolescent population, throughout the country [39]; to overcome this problem, in 2017, the meningococcal conjugate C vaccine was introduced for adolescents in the National Immunization Program, switching to meningococcal conjugate ACWY vaccine in 2020 [40].

Of note, our study period analyses should be interpreted with caution due to the increase in meningitis surveillance over time, with increasing use of PCR for meningitis diagnosis, or to the overrepresentation of regions that are more likely to send isolates to the State Central Laboratories. This resulted in increased notification in all age groups, while during the pre-vaccination period, no similar changes in the surveillance system were in place [9,41,42]. Therefore, our results might underestimate the true impact of PCV10 in children targeted to receive vaccination but overestimate the incidence in age groups not eligible to be vaccinated.

Our study has some limitations, mainly due to the use of secondary data sources. It should be taken into account that SINAN, as a passive surveillance system, may underestimate the actual number of cases. Moreover, reporting can vary depending on the ability of the epidemiological and laboratory surveillance system in each municipality to detect, notify, investigate, and perform specific laboratory tests for the etiological diagnosis of bacterial meningitis. Although SINAN classifies meningitis by etiologic agent, around 16% of cases were classified as meningitis of unspecified cause in the analyzed period. Therefore, some confirmed meningitis cases in SINAN may be incorrectly classified.

On the other hand, the strengths of the study include a long-term period of vaccine implementation country-wide, allowing an assessment of changes in the disease epidemiology before and after vaccine introduction. Moreover, all suspected cases reported to SINAN must be investigated and classified in the system as confirmed or discarded cases in accordance with National Epidemiological Surveillance criteria. Although the suspected cases that were inconclusive may represent real cases and a source of underreporting, they represented only 5% of all suspected cases, reinforcing the representativeness of the database and the robustness of the surveillance system.

A substantial reduction in invasive pneumococcal disease has been reported in some countries during the COVID-19 pandemic [43,44]. The pandemic has heavily impacted Brazil with the disruption of health systems, which makes it somewhat difficult to interpret 2020–2022 SINAN data [45]. Further studies are required to monitor pneumococcal meningitis trends in the post-pandemic era with its gradual “return to normal” and the reemergence of RSV and influenza virus circulation that can act as cofactors in pneumococcal pathogenesis [46,47]. Additionally, continuous monitoring of changes in serotype distribution is necessary to understand the pneumococcal serotype distribution across different age groups in the Brazilian population and how it impacts clinical outcomes.

## 5. Conclusions

This study provided a comprehensive assessment of pneumococcal meningitis trends over 13 years, most of them after the introduction of PCV10 in Brazil. The beneficial effect of PCV10 in reducing pneumococcal meningitis incidence has been shown in the target population eligible for vaccination in the National Immunization Program. In the pediatric population, a 56.5% reduction in the pneumococcal meningitis incidence in the post-PCV10 period. In contrast, a significant increasing trend of 2% per year was seen in older adults over the study period, which may reflect high rates of pneumococcal infection in the community, a lack of herd protection in adults of vaccines in use for the pediatric population, and no public routine pneumococcal immunization program targeting older adults.

Findings from this study contribute to the evidence supporting the direct benefits of PCV on pneumococcal meningitis among the pediatric population. The significant increasing trend of pneumococcal meningitis in older adults in the post-PCV10 period suggests a lack of indirect protection from routine pediatric vaccination. These findings highlight the value of expanding pneumococcal vaccination policies to include vaccines that provide better indirect protection from children to adults and broadening vaccination to older adults.

## Figures and Tables

**Table 1:** Number of cases and case fatality rate due to bacterial meningitis by etiology, Brazil, 2007–2019.

	Meningococcal Meningitis ^a^	Pneumococcal Meningitis	*Haemophilus* Meningitis	Meningitis by Other Bacteria	Total
Cases, n (%)					
All ages	26,077	13,837	1675	39,614	81,203
<1 year	3267 (25.5)	1868 (14.6)	571 (4.5)	7105 (55.5)	12,811 (100)
1–4 years	4957 (38.7)	1181 (9.2)	416 (3.2)	4919 (38.4)	11,473 (100)
5–17 years	8005 (62.5)	1957 (15.3)	240 (1.9)	7807 (60.9)	18,009 (100)
18–49 years	7391 (57.7)	4811 (37.6)	259 (2.0)	11,338 (88.5)	23,799 (100)
50–59 years	1144 (8.9)	1895 (14.8)	90 (0.7)	3399 (26.5)	6528 (100)
≥60 years	944 (7.4)	1948 (15.2)	81 (0.6)	4531 35.4)	7504 (100)
Missing age	369 (2.9)	177 (1.4)	18 (0.1)	515 (4.0)	1079 (100)
Case fatality rate, %					
All ages	21.1%	29.1%	15.1%	12.8%	18.3%
<1 year	22.8%	32.7%	21.0%	9.6%	16.8%
1–4 years	19.5%	28.1%	13.5%	4.6%	13.8%
5–17 years	16.6%	16.7%	9.6%	6.2%	12.0%
18–49 years	23.1%	26.4%	10.4%	14.2%	19.4%
50–59 years	27.3%	35.4%	15.6%	22.1%	26.8%
≥60 years	35.5%	38.6%	12.3%	27.1%	30.9%

^a^ Meningococcal disease is caused by *N. meningitidis* and includes meningococcemia, meningococcal meningitis, meningococcal meningitis with meningococcemia.

**Table 2:** Trends in the annual incidence rates of pneumococcal and bacterial meningitis according to the study period and age group using joinpoint regression analysis.

	Per Time Trend Inflection		Entire Period 2007–2019	Post-Vaccination Period 2011–2019	Late Post-Vaccination Period 2016–2019
	Year	APC (95% CI)	AAPC (95% CI)	AAPC (95% CI)	AAPC (95% CI)
***Pneumococcal meningitis***					
All ages	2007–2011	1.1 (−3.6; 6.0)	−1.2 (−3.8; 1.5)	−2.3 (−5.7; 1.3)	2.4 (−2.7; 7.8)
	2011–2015	−6.8 (−13.8; 0.8)			
	2015–2019	2.4 (−2.7; 7.8)			
<1 year	2007–2009	−2.7 (−21.8; 21)	−9.2 * (−14.5; −3.5)	−8.0 * (−11.5; −4.3)	−6.2 * (−10.4; −1.7)
	2009–2012	−19.6 (−38.1; 4.4)			
	2012–2019	−6.2 * (−10.4; −1.7)			
1–4 years	2007–2010	1.1 (−12.4; 16.6)	−4.7 (−11.5; 2.6)	−4.3 (−11.2; 3.1)	2.9 (−3.7; 10.0)
	2010–2013	−22.9 (−45.0; 7.9)			
	2013–2019	2.9 (−3.7; 10.0)			
5–17 years	2007–2019	−3.2 * (−5.5; −0.9)	−3.2 * (−5.5; −0.9)	−3.2 * (−5.5; −0.9)	−3.2 * (−5.5; −0.9)
18- 49 years	2007–2019	0.2 (−1.6; 1.9)	0.2 (−1.6; 1.9)	0.2 (−1.6; 1.9)	0.2 (−1.6; 1.9)
50–59 years	2007–2019	0.3 (−1.7; 2.3)	0.3 (−1.7; 2.3)	0.3 (−1.7; 2.3)	0.3 (−1.7; 2.3)
≥60 years	2007–2019	2.0 * (0.2; 3.9)	2.0 * (0.2; 3.9)	2.0 * (0.2; 3.9)	2.0 * (0.2; 3.9)
***Bacterial meningitis* ^a^**					
All ages	2007–2011	−2.1 (−4.4; 0.2)	−4.7 * (−6.0; −3.4)	−6.0 * (−7.7; −4.2)	−1.0 (−5.6; 3.8)
	2011–2016	−8.8 * (−11.2; −6.3)			
	2016–2019	−1.0 (−5.6; 3.8)			
<1 year	2007–2009	0.7 (−12.0; 15.1)	−5.3 * (−8.5; −1.9)	−4.6 * (−6.6; −2.5)	−3.2 * (−5.5; −0.9)
	2009–2012	−13.5 (−25.5; 0.5)			
	2012–2019	−3.2 * (−5.5; −0.9)			
1–4 years	2007–2010	−3.8 (−12.8; 6.2)	−6.9 * (−10.8; −2.8)	−6.9 * (−11.3; −2.3)	−1.4 (−7.9; 5.5)
	2010–2014	−15.4 * (−25.4; −4.0)			
	2014–2019	−1.4 * (−7.9; 5.5)			
5–17 years	2007–2012	−1.7 (−4.5; 1.2)	−6.9 * (−9.7; −4.1)	−9.4 * (−13.2; −5.4)	−3.3 (−12.6; 7.0)
	2012–2016	−15.5 * (−22.2; −8.2)			
	2016–2019	−3.3 (−12.6; 7.0)			
18–49 years	2007–2012	2.1 (−1.3; 5.7)	−2.3 (−5.0; 0.5)	−4.4 * (−8.1; −0.6)	0.9 (−7.5; 10.2)
	2012–2016	−9.8 * (−16.7; −2.3)			
	2016–2019	0.9 (−7.5; 10.2)			
50–59 years	2007–2011	3.7 (−2.0; 9.7)	−1.4 (−3.3; 0.5)	−3.9 * (−5.6; −2.1)	−3.9 * (−5.6; −2.1)
	2011–2019	−3.9 * (−5.6; −2.1)			
≥60 years	2007–2019	−0.5 (−1.8; 0.7)	−0.5 (−1.8; 0.7)	−0.5 (−1.8; 0.7)	−0.5 (−1.8; 0.7)

APC: annual percentage change; AAPC: average annual percentage change. * *p*-value < 0.05. ^a^ Bacterial meningitis includes all cases of meningococcal disease, *Haemophilus* meningitis, pneumococcal meningitis, and other bacterial meningitis excluding tuberculous meningitis.

**Table 3:** Trends in the annual case fatality rate of pneumococcal and bacterial meningitis according to the study period and age group using joinpoint regression analysis.

	Per Time Trend Inflection		Entire Period 2007–2019	Post-Vaccination Period 2011–2019	Late Post-Vaccination Period 2016–2019
	Period	APC (95% CI)	AAPC (95% CI)	AAPC (95% CI)	AAPC (95% CI)
***Pneumococcal meningitis***					
All ages	2007–2019	−0.1 (−1.0; 0.7)	−0.1 (−1.0; 0.7)	−0.1 (−1.0; 0.7)	−0.1 (−1.0; 0.7)
<1 year	2007–2019	0.2 (−2.7; 3.3)	0.2 (−2.7; 3.3)	0.2 (−2.7; 3.3)	0.2 (−2.7; 3.3)
1–4 years	2007–2019	−1.3 (−4.5; 2.0)	−1.3 (−4.5; 2.0)	−1.3 (−4.5; 2.0)	−1.3 (−4.5; 2.0)
5–17 years	2007–2019	−0.1 (−2.7; 2.9)	−0.1 (−2.7; 2.9)	−0.1 (−2.7; 2.9)	−0.1 (−2.7; 2.9)
18–49 years	2007–2019	−0.6 (−2.4; 1.2)	−0.6 (−2.4; 1.2)	−0.6 (−2.4; 1.2)	−0.6 (−2.4; 1.2)
50–59 years	2007–2019	−0.1 (−2.2; 1.9)	−0.1 (−2.2; 1.9)	−0.1 (−2.2; 1.9)	−0.1 (−2.2; 1.9)
≥60 years	2007–2019	−1.7 * (−3.1; −0.4)	−1.7 * (−3.1; −0.4)	−1.7 * (−3.1; −0.4)	−1.7 * (−3.1; −0.4)
***Bacterial meningitis* ^a^**					
All ages	2007–2009	6.9 (−0.1; 14.2)	1.0 * (0.0; 2.1)	−0.1 (−0.6; 0.5)	−0.1 (−0.6; 0.5)
	2009–2019	−0.1 (−0.6; 0.5)			
<1 year	2007–2019	−1.8 * (−3.2; −0.5)	−1.8 * (−3.2; −0.5)	−1.8 * (−3.2; −0.5)	−1.8 * (−3.2; −0.5)
1–4 years	2007–2009	17.7 (−1.4; 40.6)	−1.2 (−5.8; 3.6)	−1.8 (−5.0; 1.4)	0.2 (−3.7; 4.3)
	2009–2012	−15.0 (−30.4; 3.8)			
	2012–2019	0.2 (−3.7; 4.3)			
5–17 years	2007–2009	13.2 (−6.6; 37.3)	2.1 (−0.9; 5.2)	0 (−1.7; 1.8)	0 (−1.7; 1.8)
	2009–2019	0 (−1.7; 1.8)			
18–49 years	2007–2019	−0.3 (−1.2; 0.6)	−0.3 (−1.2; 0.6)	−0.3 (−1.2; 0.6)	−0.3 (−1.2; 0.6)
50–59 years	2007–2019	−0.5 (−1.7; 0.8)	−0.5 (−1.7; 0.8)	−0.5 (−1.7; 0.8)	−0.5 (−1.7; 0.8)
≥60 years	2007–2019	−0.3 (−1.6; 0.9)	−0.3 (−1.6; 0.9)	−0.3 (−1.6; 0.9)	−0.3 (−1.6; 0.9)

APC: annual percent change; AAPC: average annual percent change. * *p*-value <0.05. ^a^ Bacterial meningitis includes all cases of meningococcal disease, *Haemophilus* meningitis, pneumococcal meningitis, and other bacterial meningitis excluding tuberculous meningitis.

## Data Availability

All data reported were obtained from DATASUS site (http://www2.datasus.gov.br/DATASUS/index.php (accessed on 14 July 2021)).

## Supplementary Materials

The following supporting information can be downloaded at: https://www.mdpi.com/article/10.3390/vaccines11081279/s1, Table S1: National annual vaccination coverage rate, Brazil, 2007–2019; Table S2: Distribution of meningitis cases using SINAN etiology classification, Brazil, 2007–2019; Table S3: Annual incidence rates of pneumococcal and bacterial meningitis per 100,000 inhabitants, according to age group and study year, Brazil, 2007–2019; Table S4: Mean pneumococcal and bacterial meningitis incidence rates in the pre-PCV10 (2007–2009) and post-PCV10 (2011–2019) vaccination periods, Brazil; Table S5: Mean pneumococcal and bacterial meningitis case fatality rate in the overall study period and the pre-PCV10 (2007–2009) and post-PCV10 (2011–2019) vaccination periods, Brazil.
