# Supplementary material for: Trends in Pneumococcal and Bacterial Meningitis in Brazil from 2007 to 2019
Source: Vaccines (Basel). 2023 Jul 25;11(8):1279. doi: 10.3390/vaccines11081279 (PMC10459388; doi:10.3390/vaccines11081279)
Supplement: Supplementary file 1 [file vaccines-11-01279-s001.zip › vaccines-2509616-supplementary.pdf]

vaccines

MDPI

Article

Supplementary Materials:

Table S1. National annual vaccination coverage rate, Brazil, 2007–2019

Calendar year	PCV10	Meningococcal C	*Haemophilus influenzae*
2007	NA	NA	100.0
2008	NA	NA	97.6
2009	NA	NA	100.0
2010	24.0	26.9	97.7
2011	81.7	100.0	97.7
2012	88.4	96.2	93.82
2013	93.6	99.7	95.89
2014	93.5	96.4	94.85
2015	94.2	98.2	96.3
2016	95.0	91.7	89.27
2017	92.2	87.4	84.24
2018	95.3	88.5	88.49
2019	89.1	87.4	70.76
2011–2019	91.4	94.5	80.66

PCV10: 10-valent pneumococcal conjugate vaccine; NA, not available (in this year, this vaccine had not been introduced in the National Immunization Program yet).

Note: These vaccination coverages considered fully vaccinated children according to the official vaccine schedule. Vaccination coverage was based on administrative data. In some cases, the vaccination coverage exceeded 100%, due to population estimation errors. When this occured, we considered the coverage to be 100%. **Source:** *Sistema de Informação do Programa Nacional de Imunizações.* Brazilian Ministry of Health, 2022 [1].

Table S2. Distribution of meningitis cases using SINAN etiology classification, Brazil, 2007–2019.

Etiology	N	%
Meningococcal disease*	26,077	10.1%
Meningitis by other bacteria*	39,614	15.3%
Pneumococcal meningitis*	13,837	5.3%
Haemophilus meningitis*	1,675	0.6%
Tuberculous meningitis	4,681	1.8%
Unspecified meningitis	41,645	16.1%
Aseptic meningitis	119,266	46.0%
Meningitis due to another etiology	10,004	3.9%
Unspecified etiological agent	2,266	0.9%
	259,065	100%

* In this study, bacterial meningitis included meningococcal disease (meningococcemia, meningococcal meningitis, and meningococcal meningitis with meningococcemia), meningitis by other bacteria, *Haemophilus* meningitis and pneumococcal meningitis. Tuberculous meningitis was not included as bacterial meningitis in this analysis due to its subacute course and specific epidemiology.

Table S3. Annual incidence rates of pneumococcal and bacterial meningitis per 100,000 inhabitants, according to age group and study year, Brazil, 2007–2019.

	2007	2008	2009	2010	2011	2012	2013	2014	2015	2016	2017	2018	2019
	**Rate (CI 95%)**	**Rate (CI 95%)**	**Rate (CI 95%)**	**Rate (CI 95%)**	**Rate (CI 95%)**	**Rate (CI 95%)**	**Rate (CI 95%)**	**Rate (CI 95%)**	**Rate (CI 95%)**	**Rate (CI 95%)**	**Rate (CI 95%)**	**Rate (CI 95%)**	**Rate (CI 95%)**
umococcal meningitis													
ges	0.6 (0.6, 0.6)	0.6 (0.6, 0.6)	0.6 (0.5, 0.6)	0.6 (0.6, 0.6)	0.6 (0.6, 0.7)	0.6 (0.5, 0.6)	0.5 (0.5, 0.6)	0.5 (0.5, 0.5)	0.5 (0.4, 0.5)	0.5 (0.4, 0.5)	0.5 (0.5, 0.5)	0.5 (0.5, 0.5)	0.5 (0.5, 0.5)
ear	8.6 (7.6, 9.7)	8.1 (7.1, 9.2)	7.9 (6.9, 8.9)	6.7 (5.8, 7.8)	5.4 (4.6, 6.3)	3.9 (3.2 4.7)	4.1 (3.4, 4.9)	4.0 (3.3, 4.8)	2.9 (2.3, 3.5)	3.2 (2.6, 4.0)	3.0 (2.4, 3.7)	2.9 (2.3, 3.6)	2.8 (2.2, 3.4)
4 years	1.0 (0.9, 1.2	1.2 (1.0, 1.4)	1.0 (0.9, 1.2)	1.1 (1.0, 1.3)	0.9 (0.7, 1.1)	0.7 (0.6, 0.9)	0.5 (0.4, 0.7)	0.5 (0.4, 0.7)	0.5 (0.4, 0.6)	0.6 (0.5, 0.7)	0.6 (0.4, 0.7)	0.6 (0.5, 0.7)	0.7 (0.5, 0.8)
17 years	0.4 (0.4, 0.5)	0.4 (0.3, 0.4)	0.4 (0.3, 0.5)	0.5 (0.4, 0.5)	0.5 (0.4, 0.5)	0.4 (0.4, 0.5)	0.4 (0.3, 0.4)	0.3 (0.3, 0.4)	0.3 (0.3, 0.4)	0.2 (0.2, 0.3)	0.3 (0.3, 0.4)	0.3 (0.2, 0.3)	0.4 (0.3, 0.4)
49 years	0.3 (0.3, 0.4)	0.4 (0.3, 0.4)	0.3 (0.3, 0.4)	0.4 (0.3, 0.4)	0.4 (0.4, 0.5)	0.4 (0.4, 0.5)	0.4 (0.4 0.5)	0.4 (0.3, 0.4)	0.4 (0.3, 0.4)	0.3 (0.3 0.4)	0.4 (0.3, 0.4)	0.4 (0.3, 0.4)	0.4 (0.4, 0.4)
59 years	0.6 (0.5, 0.7)	0.7 (0.6, 0.8)	0.6 (0.5, 0.7)	0.8 (0.7, 0.9)	0.8 (0.7, 1.0)	0.8 (0.7, 1.0)	0.8 (0.7, 1.0)	0.7 (0.6, 0.9)	0.6 (0.5, 0.8)	0.7 (0.6, 0.9)	0.7 (0.6, 0.8)	0.8 (0.7, 0.9)	0.6 (0.5, 0.7)
years	0.6 (0.5, 0.7)	0.5 (0.4, 0.6)	0.5 (0.5, 0.7)	0.6 (0.5, 0.7)	0.7 (0.6, 0.8)	0.7 (0.6, 0.8)	0.6 (0.5, 0.8)	0.6 (0.5, 0.7)	0.6 (0.5, 0.7)	0.7 (0.6, 0.8)	0.8 (0.7, 0.9)	0.7 (0.6, 0.8)	0.6 (0.5, 0.7)
terial meningitis [a]													
ges	4.1 (4.0, 4.2)	4.0 (4.0, 4.1)	3.9 (3.8, 4.0)	3.9 (3.8, 4.0)	3.7 (3.7, 3.8)	3.5 (3.4, 3.6)	3.1 (3.0, 3.2)	2.8 (2.7, 2.9)	2.6 (2.5, 2.6)	2.3 (2.3, 2.4)	2.4 (2.4, 2.5)	2.5 (2.4, 2.5)	2.2 (2.1, 2.3)
ear	47.3 (44.8, 49.8)	45.0 (42.6, 47.5)	46.8 (44.3, 49.3)	42.7 (40.4, 45.2)	34.6 (32.5, 36.8)	29.7 (27.8, 31.8)	29.5 (27.5, 31.5)	28.6 (26.7, 30.6)	28.6 (26.7, 30.6)	26.4 (24.5, 28.3)	25.4 (23.6, 27.3)	27.1 (25.2, 29.0)	22.6 (20.9, 24.4)
4 years	11.5 (10.9, 12.1)	11.2 (11.0, 11.8)	10.4 (9.9, 11.0)	10.1 (9.5, 10.7)	9.0 (8.4, 9.5)	7.8 (7.3, 8.3)	5.6 (5.2, 6.0)	5.6 (5.1, 6.0)	5.1 (4.7, 5.5)	4.6 (4.2, 5.0)	5.5 (5.1, 6.0)	5.2 (4.8, 5.6)	4.7 (4.3, 5.1)
17 years	4.6 (4.5, 4.9)	4.2 (4.0, 4.3)	4.2 (4.0, 4.4)	4.2 (4.1, 4.4)	4.2 (4.0, 4.4)	4.1 (4.0, 4.3)	3.4 (3.2, 3.6)	3.0 (2.8, 3.2)	2.4 (2.2, 2.5)	2.0 (1.9, 2.2)	2.2 (2.1, 2.4)	1.9 (1.8, 2.1)	1.8 (1.7, 2.0)
49 years	1.9 (1.8, 2.0)	2.2 (2.1, 2.3)	2.1 (2.0, 2.2)	2.1 (2.0, 2.2)	2.2 (2.1, 2.3)	2.2 (2.1, 2.3)	2.0 (1.9, 2.1)	1.8 (1.7, 1.9)	1.6 (1.6, 1.7)	1.5 (1.4, 1.5)	1.5 (1.4, 1.6)	1.6 (1.5, 1.7)	1.5 (1.4, 1.5)
59 years	2.5 (2.2, 2.7)	2.7 (2.4, 2.9)	2.5 (2.2, 2.7)	2.8 (2.5, 3.0)	2.8 (2.6, 3.1)	2.9 (2.2, 3.1)	2.8 (2.5, 3.0)	2.5 (2.3, 2.7)	2.3 (2.1, 2.5)	2.2 (2.0, 2.4)	2.3 (2.1, 2.5)	2.3 (2.1, 2.5)	2.1 (1.9, 2.3)
years	2.3 (2.1, 2.5)	2.6 (2.4, 2.8)	2.3 (2.1, 2.5)	2.5 (2.3, 2.8)	2.9 (2.7, 3.1)	2.6 (2.4, 2.9)	2.4 (2.2, 2.6)	2.4 (2.2, 2.6)	2.4 (2.2, 2.6)	2.5 (2.3, 2.7)	2.5 (2.3, 2.6)	2.6 (2.4, 2.8)	2.1 (2.0, 2.3)

[a] Bacterial meningitis includes meningococcemia, meningococcal meningitis, meningococcal meningitis with meningococcemia, other bacterial meningitis, *Haemophilus* meningitis and pneumococcal meningitis.

Table S4. Mean pneumococcal and bacterial meningitis incidence rates in the pre-PCV10 (2007–2009) and post-PCV10 (2011–2019) vaccination periods, Brazil.

	Overall period 2007–2019		Pre-PCV10 period 2007–2009		Post-PCV10 period 2011–2019		% change	95% CI
	Mean	95% CI	Mean	95% CI	Mean	95% CI		
Pneumococcal meningitis								
All ages	0.5	0.5, 0.5	0.6	0.6, 0.6	0.5	0.5, 0.5	−12.3	−15.7, −8.7
<1 year	4.9	4.7, 5.1	8.2	7.9, 8.8	3.6	3.3, 3.8	−56.5	−60.6, −52.0
1 to 4 years	0.8	0.7, 0.8	1.1	1.0, 1.2	0.6	0.6, 0.7	−44.4	−51.0, −36.8
5 to 17 years	0.4	0.3, 0.4	0.4	0.4, 0.4	0.3	0.3, 0.4	−12.7	−21.3, −3.1
18 to 49 years	0.4	0.4, 0.4	0.4	0.3, 0.4	0.4	0.4, 0.4	10.7	3.1, 18.9
50 to 59 years	0.7	0.7, 0.8	0.6	0.6, 0.7	0.7	0.7, 0.8	16.2	3.0, 31.4
≥60 years	0.6	0.6, 0.7	0.5	0.5, 0.6	0.7	0.6, 0.7	25.3	10.8, 42.1
Bacterial meningitis [a]								
All ages	3.1	3.1, 3.2	4.0	4.0, 4.1	2.8	2.8, 2.8	−30.7	−31.7, −29.6
<1 year	33.4	32.8, 34.0	46.4	45.0, 47.8	28.0	27.4, 28.7	−39.5	−41.8, −37.2
1 to 4 years	7.4	7.3, 7.6	11.0	10.7, 11.4	5.9	5.7, 6.0	−46.8	−48.9, −44.6
5 to 17 years	3.3	3.3, 3.4	4.3	4.2, 4.5	2.8	2.8, 2.9	−35.2	−37.2, −33.0
18 to 49 years	1.8	1.8, 1.9	2.0	2.00, 2.1	1.8	1.7, 1.8	−14.2	−16.8, −11.6
50 to 59 years	2.5	2.4, 2.5	2.5	2.4, 2.7	2.4	2.4, 2.5	−4.0	−9.7, 2.1
≥60 years	2.5	2.4, 2.5	2.4	2.3, 2.5	2.5	2.4, 2.5	3.2	−2.7, 9.6

[a] Bacterial meningitis includes meningococcemia, meningococcal meningitis, meningococcal meningitis with meningococcemia, other bacterial meningitis, *Haemophilus* meningitis and pneumococcal meningitis.

Table S5. Mean pneumococcal and bacterial meningitis case fatality rate in the overall study period and the pre-PCV10 (2007–2009) and post-PCV10 (2011–2019) vaccination periods, Brazil.

	Overall period 2007–2019		Pre-PCV period 2007–2009		Post-PCV period 2011–2019		% change	95% CI
	**Mean**	**95% CI**	**Mean**	**95% CI**	**Mean**	**95% CI**		
Pneumococcal meningitis								
All ages	29.1	28.2 , 30.0	29.6	27.8 , 31.5	28.8	27.7 , 29.9	−2.8	−9.7 , 4.7
<1 year	32.7	30.2 , 35.4	34.3	30.2 , 38.9	31.5	28.0 , 35.3	−8.2	−22.6 , 9.1
1 to 4 years	28.1	25.2 , 31.3	29.8	24.7 , 35.6	25.2	21.5 , 29.4	−15.4	−33.6 , 8.15
5 to 17 years	16.7	15.0 , 18.6	18.1	14.6 , 22.2	16.1	14.0 , 18.5	−11.0	−30.8 , 15.0
18 to 49 years	26.4	25.0 , 27.9	26.5	23.3 , 29.9	26.4	24.7 , 28.2	−0.2	−13.1 , 15.0
50 to 59 years	35.4	32.7 , 38.1	33.6	27.7 , 40.5	35.5	32.4 , 38.7	5.4	−14.4 , 30.6
≥60 years	38.6	35.8 , 41.4	42.5	35.6 , 50.5	37.9	34.8 , 41.1	−11.0	−26.5 , 8.5
Bacterial meningitis[a]								
All ages	18.3	18.0 , 18.6	17.48	16.9 , 18.0	18.59	18.2 , 19.0	6.4	2.5 , 10.4
<1 year	16.8	16.1 , 17.6	18.14	16.9 , 19.5	15.84	15.0 , 16.8	−12.7	−20.4 , −4.1
1 to 4 years	13.8	13.1 , 14.5	15.92	14.7 , 17.2	11.88	11.0 , 12.8	−25.4	−32.9 , −16.9
5 to 17 years	12.0	11.5 , 12.5	10.95	10.1 , 11.8	12.44	11.8 , 13.1	13.6	3.2 , 25.1
18 to 49 years	19.4	18.8 , 20.0	19.03	17.9 , 20.2	19.54	18.9 , 20.2	2.7	−4.2 , 10.1
50 to 59 years	26.8	25.5 , 28.1	25.97	23.3 , 28.9	26.93	25.5 ; 28.5	3.7	−8.0 ; 17.2
≥60 years	30.9	29.7 ; 32.2	31.3	28.4 ; 34.5	31.0	29.5 ; 32.5	−0.9	−10.8 ; 10.4

[a] Bacterial meningitis includes meningococcemia, meningococcal meningitis, meningococcal meningitis with meningococcemia, other bacterial meningitis, *Haemophilus* meningitis and pneumococcal meningitis.References.

Refreences

1. Informações de saúde: Immunizações-cobertura-brasil. 2023 06/25/2023]. Available from: http://tabnet.datasus.gov.br/cgi/tabcgi.exe?pni/cnv/cpniuf.def
